# Indicators of clinical performance in monitoring soft tissue sarcoma management: a population-based perspective

**DOI:** 10.3389/fmed.2023.1226090

**Published:** 2023-08-08

**Authors:** Massimo Rugge, Alessandra Buja, Saveria Tropea, Giovanni Girardi, Claudia Cozzolino, Manuel Zorzi, Antonella Vecchiato, Antonella Stefano, Paolo Del Fiore, Antonella Brunello, Alessandra Brazzale, Marta Sbaraglia, Angelo Paolo Dei Tos, Vincenzo Baldo, Patrizia Benini, Alberto Bortolami, Marco Rastrelli, Luigi Dall'Olmo, Carlo Riccardo Rossi, Simone Mocellin

**Affiliations:** ^1^Pathology and Cytopathology Unit, Department of Medicine—DIMED, University of Padua, Padua, Italy; ^2^Veneto Tumor Registry, Azienda Zero, Padua, Italy; ^3^Department of Cardiac, Thoracic, Vascular Sciences, and Public Health, University of Padua, Padua, Italy; ^4^Soft-Tissue, Peritoneum and Melanoma Surgical Oncology Unit, Veneto Institute of Oncology IOV—IRCCS, Padua, Italy; ^5^Department of Women’s and Children’s Health—SDB, University of Padua, Padua, Italy; ^6^Medical Oncology 1 Unit, Veneto Institute of Oncology IOV—IRCCS, Padua, Italy; ^7^Department of Statistical Sciences, University of Padua, Padua, Italy; ^8^Department of Pathology, Azienda Ospedale Università Padova, Padua, Italy; ^9^Directorate General, Veneto Institute of Oncology IOV—IRCCS, Padua, Italy; ^10^Rete Oncologica Veneta ROV, Padova, Italy; ^11^Department of Surgery, Oncology and Gastroenterology—DISCOG, University of Padua, Padua, Italy

**Keywords:** clinical indicators, monitoring quality of care, quality assurance, clinical pathways, quality of health care, health care evaluation, soft tissue sarcoma

## Abstract

**Background:**

Soft tissue sarcomas (STS) are rare malignancies which prognosis varies significantly by primary site, histological subtype, and tumor stage. Their low incidence, and the complexity of their clinico-pathological characteristics demand standardized, cancer-tailored diagnostics and therapies managed at high-volume, multidisciplinary care centers. This study evaluates the quality of STS management in north-east Italy (Veneto Region) through a list of *ad hoc* defined clinical indicators.

**Methods:**

This population-based study concerns all incident cases of STS in 2018 (214 cases) recorded in the adult population censored by the Veneto’s regional Cancer Registry. Based on the international literature, a multidisciplinary working group of experts identified a set of indicators for monitoring the quality of diagnostic, therapeutic, and end-of-life clinical interventions. The quality of care was assessed by comparing the reference thresholds with the indicators’ values achieved in clinical practice.

**Results:**

Diagnostic procedures showed poor adherence to the thresholds, with a low percentage of histological diagnoses validated by a second opinion. The indicators relating to the surgical treatment of superficial, small, low-grade STS, or of medium, high-grade STS of the head–neck, trunk, or limbs were consistent with the thresholds, while for intermediate, high-grade (large-sized, deep) and retroperitoneal STS they fell significantly below the thresholds.

**Conclusion:**

A critical evaluation of the clinical indicators allowed to uncover the procedures needing corrective action. Monitoring clinical care indicators improves cancer care, confirms the importance of managing rare cancers at highly specialized, high-volume centers, and promotes the ethical sustainability of the healthcare system.

## Introduction

Soft tissue sarcomas (STS) account for less than 2% of all malignant tumors in adults. They include a wide range of different malignancies (1–3), classified according to their cell lineage (adipocytic, chondro-osseous, fibroblastic or myofibroblastic, fibro-histiocytic, nerve sheath tumors, pericytic, skeletal muscle, smooth muscle, vascular, tumors of uncertain differentiation, and undifferentiated/unclassified sarcoma) (4, 5). Inside these main lineage classes, histological phenotypes, immunohistochemical patterns, and molecular profiling distinguish more than 100 subtypes that differ significantly in their clinical outcome (6). In western countries, the mean 5-year STS overall survival rate in adults reaches approximatively 65%; however, this value varies considerably—from 80 to 15%–according to the different considered histotypes, neoplastic stages, or operative contexts (7).

In cancer care, adopting standardized diagnostic and therapeutic strategies enables care quality to be monitored consistently, and healthcare system efficiency to be critically addressed. Both these actions are essential to improving patient outcomes and optimizing the allocation of resources (8–11). Variability in cancer patient management may stem from issues relating to healthcare management policy and/or to the volume of clinical experience. For low-incident cancers like STS, this means that patients should be referred to specialized centers, as high-volume clinical experience is a well-recognized promoter of good clinical practice (8–14).

Quality of patient care can be reliably monitored by means of “indicators” addressing a center’s performance, and the outcomes of the diagnostic-therapeutic pathway adopted. In oncology, as in other clinical specialties, quality indicators are useful to both identify and quantify: (i) the appropriateness of diagnostic procedures; (ii) the efficacy of anticancer therapies and surgical treatments; (iii) the critical areas most requiring corrective actions; and (iv) the sustainability and relative priority of investments directed toward oncological cares (9, 10).

This population-based study critically addresses the clinical management of STS patients’ resident in the Veneto region of Italy in the year 2018. Quality of care was ranked thon the strength of a set of internationally-acknowledged clinical indicators selected by a multidisciplinary regional working group (RWG) of specialists with expertise in soft tissue malignancies.

## Materials and methods

### Context

The Italian National Health System is a public service supported primarily by general taxation and it is managed on a regional basis. Its policies are grounded on the fundamental values of universality, free access, freedom of choice, pluralism in provision, and equity.

In 2015, the Veneto’s Regional Oncology Network, comprising Veneto Oncology Institute, a center included in the European network for Rare adult solid Cancer (EURACAN), produced a comprehensive document detailing the clinical procedures to be applied in each step of the clinical management of STS patients, from their initial diagnosis to their end-of-life care (15, 16). The Veneto Oncology Clinical Pathway’s document was based on current national/international literature (17–20).

### Clinical data

Data were collected from the population-based Veneto Cancer Registry, a high-resolution database which covers the population of the whole region (4.9 million residents), and from the regional health service records. The present study concerns all incident cases of STS recorded by the Registry in the year 2018. Recording procedures rely on various informative sources, such as pathology reports, clinical charts, death certificates, and health service administrative records. The variables available for STS include: age and sex; tumor site (limbs, head, trunk, and retroperitoneum); diameter of the primary tumor (mm); depth (superficial versus deep); histological subtype (ICD-O-3 code); tumor grade (G1, G2, G3, and GX); combined clinical-pathological TNM stage at diagnosis (I, II, III, and IV); treatments (surgical, medical, and multimodal); and status of resection margins (R0, R1, and R2). The clinical information recorded also includes: the results of diagnostic imaging [US, computerized tomography (CT), MRI, Positron Emission Tomography (PET)]; the identification code of the institution(s) delivering the treatment; and the timing of therapeutic procedures [surgery, chemotherapy (ChT); and radiation (RT)].

### Indicators

In 2021, a RWG of epidemiologists, healthcare managers, oncologists, pathologists, radiologists, radiotherapists, statisticians, and surgeons established a list of indicators to use in monitoring the quality of care provided for adult STS patients, the consistency between threshold values adopted for these indicators, and how centers performed in real-world clinical practice. All nine regional public health institutions potentially involved in STS care were included in this quality assessment (QA) project.

**Figure 1:**
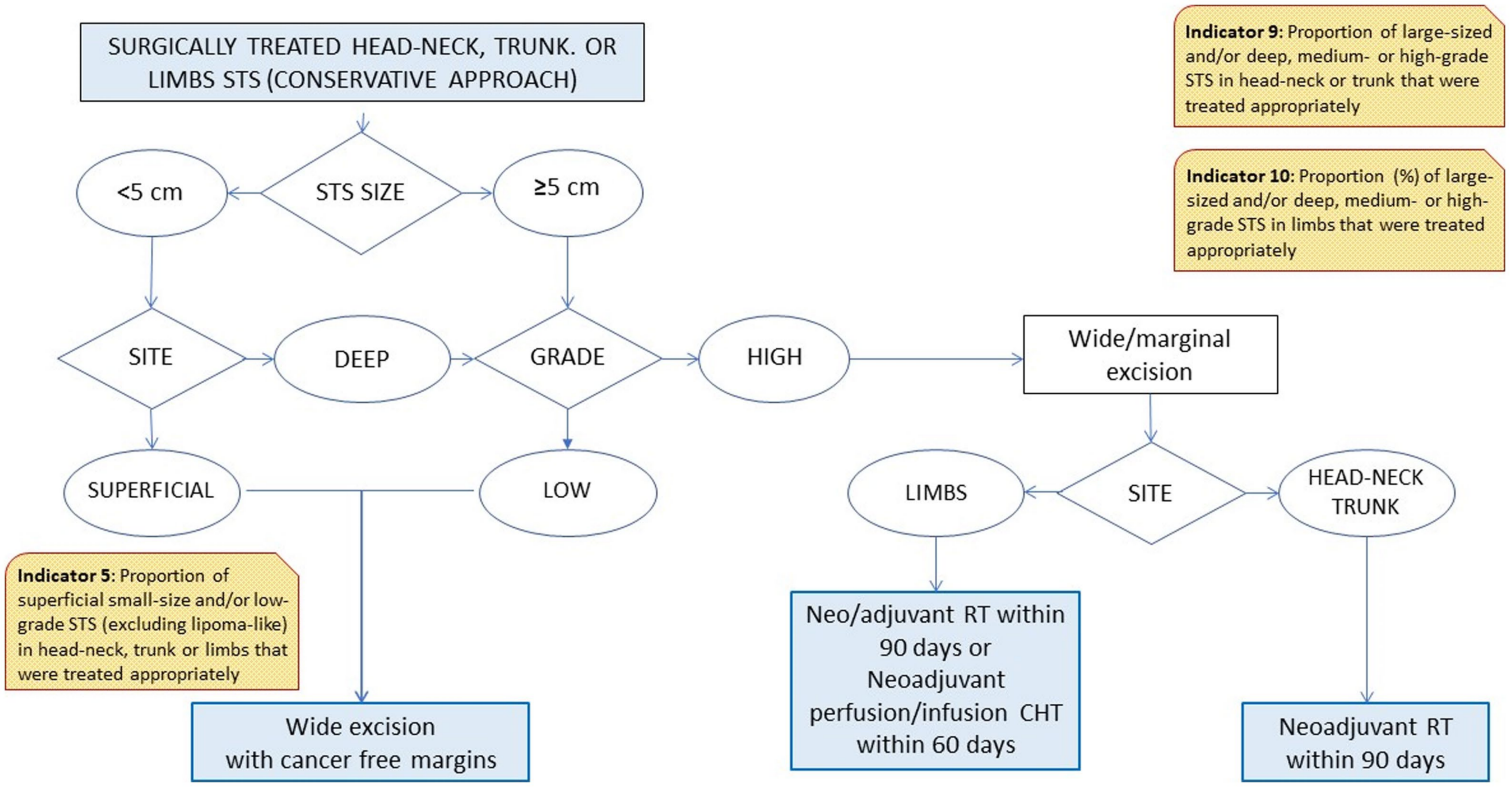
Flow chart for treatment indicators 5, 9, and 10.

Based on the different steps of a patient’s clinical management (21, 22), the RWG identified six main care provision phases, each of which was tested against a variable number of indicators (Table 1). The phases were: diagnosis (two indicators); process performance (two indicators); surgical treatments (four indicators); combined surgical and medical treatments (three indicators); medical treatments (three indicators); and end-of-life management (one indicator). A representative example of the algorithm applied to establish the appropriateness of a care pathway is shown in Figure 1. In cases where more than one type of treatment was delivered, the algorithm also assessed the consistency of the timing of multimodal therapies (e.g., pathology, imaging, and surgery; ChT, RT).

**Table 1:** Quality indicators chosen by the regional working group on soft tissue sarcoma.

Operative phase	Indicators	Threshold	Estimated percentage (95% CI)
1. Diagnosis	Proportion (%) of deep STS (any size), or superficial STS (> 5 cm) without MRI/CT before biopsy	<5%	**10.59** (5.44, 19.26)
2. Diagnosis	Proportion (%) of second opinions obtained for STS diagnostic biopsy	>90%	**45.40** (37.70, 53.10)
3. Process performance	Proportion (%) of surgical or medical treatments administered within 90 days after diagnostic biopsy	>90%	90.30 (84.04, 94.57)
4. Process performance	Proportion (%) of patients given at least one surgical treatment at non-reference STS centers in the region out of total STS patients treated surgically in Veneto	<30%	**70.59** (63.26, 77.11)
5. Surgical therapy	Proportion (%) of superficial small-size and/or low-grade STS (excluding lipoma-like) in head–neck, trunk or limbs that were treated appropriately (Figure 1)	>80%	92.98 (82.73, 97.57)
6. Surgical therapy	Proportion (%) of low-grade, lipoma-like STS in head–neck, trunk, or limb, that were treated appropriately (all types of surgery, including enucleation)	>80%	100.00 (63.54, 100.00)
7. Surgical therapy	Proportion (%) of medium- or high-grade STS of head–neck, trunk, or limbs showing clear margins after surgical treatment	>90%	**80.00** (72.06, 86.29)
8. Surgical therapy	Proportion (%) of retroperitoneal STS treated with multivisceral surgery	>80%	**36.59** (22.91, 52.46)
9. Surgical-medical therapy	Proportion (%) of large-sized and/or deep, medium- or high-grade STS in head–neck or trunk that were treated appropriately (Figure 1)	>80%	**34.62** (18.81, 54.21)
10. Surgical-medical therapy	Proportion (%) of large-sized and/or deep, medium- or high-grade STS in limbs that were treated appropriately (Figure 1)	>80%	**70.00** (51.69, 83.68)
11. Surgical-medical therapy	Proportion (%) of STS in head–neck, trunk or that were treated appropriately (cumulative value of indicators 5, 6, 9, and 10)	>80%	**75.00** (66.30, 82.18)
12. Medical therapy	Proportion (%) of medium- or high-grade STS of head–neck, trunk, or limbs, deep and > 5 cm in diameter, radically removed with conservative surgery and treated with RT within 90 days before or after surgery	>90%	**66.67** (45.76, 83.09)
13. Medical therapy	Proportion (%) of STS of limbs, deep and > 5 cm in diameter, grade G3, radically removed with conservative surgery and treated with ChT within 60 days before or after surgery	>90%	**36.36** (13.51, 66.71)
14. Medical therapy	Proportion (%) of patients withdrawn from ChT due to toxicity	No threshold	1.96 (0.10, 10.44)
15. End of life care	Proportion (%) of patients treated with ChT within 30 days before their death	<10%	**31.43** (18.25, 48.56)

Threshold values were based on the current literature. In bold, the indicators that did not reach the threshold. Estimated percentage and 95%CI calculated on data obtained from the regional health service administration (year 2018). STS, soft tissue sarcoma; MRI, magnetic resonance imaging; CT, computerized tomography; RT, radiotherapy; and ChT, chemotherapy.

## Results

In 2018, the regional population-based cancer registry (censoring the whole Veneto population of nearly 5 million) recorded 214 incident cases of adult STS. Table 2 shows the demographics and clinico-pathological profile of these STS patients.

**Table 2:** Demographics and clinico-pathological profile of the adult STS cohort.

Variable	Total STS patients: 214 (%)	
Sex	Male	124 (57.9)
	Female	90 (42.1)
Age	20–29 (M:*F* = 0:1)	1 (0.5)
Mean = 65.9 (SD = 15.3)	30–39 (M:*F* = 4:9)	13 (6.1)
Median = 67	40–49 (M:*F* = 17:8)	25(11.7)
	50–59 (M:*F* = 16:18)	34 (15.9)
	60–69 (M:*F* = 24:20)	44 (20.6)
	70–79 (M:*F* = 42:16)	58 (27.1)
	80–89 (M:*F* = 20:13)	33 (15.4)
	≥90 (M:F = 1:5)	6 (2.8)
Primary site	Limbs	81 (37.9)
	Trunk	56 (26.2)
	Retroperitoneum	50 (23.4)
	Head–neck	24 (11.2)
	Unknown	3 (1.4)
Lineage of cell differentiation	Uncertain differentiation	60 (28.0)
	Liposarcoma	55 (25.7)
	Fibroblastic/myofibroblastic sarcoma	43 (20.1)
	Leiomyosarcoma	34 (15.9)
	Vascular sarcoma	12 (5.6)
	Others	10 (4.7)
TNM stage at initial diagnosis (AJCC 7th edition)	I	46 (21.5)
	II	67 (31.3)
	III	51 (23.8)
	IV	29 (13.6)
	Unknown	21 (9.8)

Table 1 lists the 15 indicators considered, by phase of the clinical pathway: diagnostic, process performance, therapeutic (surgical and non-surgical), and end of life care. The table also shows the threshold values of the indicators established by the RWG, and the real-world values identified by the present study.

### Diagnostic phase: indicators 1–2

Pre-biopsy imaging, as recommended by the international and regional guidelines, was available for 89% of patients. The initial diagnosis of STS was supported by a second opinion in 45% of cases. Both these indicators fell below the established thresholds.

### Process performance: indicators 3–4

These two performance indicators measure whether and when the activities recommended in order to accomplish the strategic objectives of the integrated care processes actually took place (23). More than 90% of STS patients received a treatment within 90 days from their histological diagnosis (indicator 3), and 71% of patients were at least partially treated with surgical procedures at non-reference centers (indicator 4).

### Therapeutic phase (surgical and other treatments): indicators 5–14

Two of the four indicators relating to surgical therapies (Nos. 5, 6, 7, and 8) were consistent with the thresholds. Among the 2018 incident cases of retroperitoneal STS, however, only 37% were treated with multivisceral surgery (well below the threshold of >80%; indicator No. 8).

None of the indicators relating to combined therapies (surgery plus other treatments; indicators Nos. 9, 10, and 11), or medical therapies (indicators Nos. 12, 13) were consistent with the thresholds. The best performance (75 vs. a threshold of 80%) was achieved for indicator No. 11 relating to how multimodal therapies were administered for head–neck, trunk, or limb STS of any size or histological grade. The prevalence of ChT withdrawal due to toxicity was 2%.

### End of life care (indicator 15)

More than 30% of patients were given ChT within 30 days before their death.

## Discussion

This study assessed how the Veneto healthcare system performed in the clinical management of 214 incident cases of adult STS diagnosed in 2018. The evaluation was conducted with a list of *ad hoc* defined indicators concerning all the main clinical phases of STS care: diagnosis, process performance, surgical/medical treatments, and end-of-life care. The benchmark value for of the indicators established by an interdisciplinary regional group of experts was compared with the value obtained in real-world clinical practice.

### Diagnosis

A suboptimal management of the diagnostic procedures came to light as concerned both the proportion of STS cases assessed on imaging before a biopsy was performed (threshold <5%, actual practice 11%), and the proportion of histological diagnoses confirmed by a second opinion (threshold >90%, actual practice 45%).

The gross features of the tumor (e.g., site and size) and the microscopic phenotype are determinants in STS care. Diagnostic procedures chiefly involve two methods: imaging (MRI, CT, and even US or PET; indicator No. 1); and pathology (histology, immunocytochemistry, molecular biology; indicator No. 2). As a rule, however, the conclusive assessment demands a critical merging of both. The high proportion of STS patients who underwent imaging only after a biopsy (threshold <5%, actual practice 16%) is a concern because the lack of information from propaedeutic imaging data significantly limits the pathologist’s assessment. Mathoulin-Pélissier et al. (24, 25) mentioned this same inconsistency in the timing of the diagnostic procedures in 2014, in reporting a low adherence in real life to three well-established good practice criteria: (a) receiving the histological diagnosis before surgery; (b) adequacy of the histological diagnosis; and (c) multidisciplinary discussion before surgery (adherence <30%).

The low incidence of STS, and the variety of histological subtypes, significantly limit operators’ experience with its diagnosis. This results in high rates of intra/inter-observer variability (26–28). Comparing the diagnostic concordance in soft tissue and bone sarcoma, assessed at two comprehensive cancer centers, complete diagnostic consistency, partial agreement, and significant disagreement were achieved in 62.5, 26.1, and 11.4% of cases, respectively (29, 30). More recently, a European multicenter study (26) addressing the diagnostic consistency between initial and second opinions echoed the above-mentioned findings, showing that full diagnostic concordance only slightly exceeding 55% of the cases considered (824/1463), while 35 and 8% of the cases were the object of only partial agreement or complete discordance, respectively. In the light of such data, we can assume second opinions could change the initial diagnosis in about 45% of cases, meaning a potential misclassification of about 25% of cases of STS. Together with the above findings, the results of the present study underscore the importance of inter-center diagnostic networks or mandatory second expert opinions, despite the fact that the Veneto Region has an excellent, nationally and internationally recognized anatomic pathology reference center for STS. However, precisely to achieve this goal, regional governance has recently established specific legislation to support second opinion procedures.

### Process performance

The RWG’s process performance indicators measured the quality of the integrated healthcare services for STS, revealing a partial misalignment between the expected threshold values and the evidence on actual clinical patient management in 2018. The proportion of patients treated within 3 months of receiving their histological diagnosis was compliant with the 90% expected threshold. However, a high proportion of patients were not treated entirely at reference centers. The timely referral of patients with rare cancers to specialized institutions for all their care reduces the time elapsing between diagnosis and therapy, increasing the efficacy and efficiency of treatments.

The results emerging from this study point to the need for a combined strategy involving healthcare managers, physicians, and patients (or patients’ associations), to ensure that: (i) public healthcare policy promotes dedicated care strategies for low-incident cancers; (ii) healthcare institutions disseminate consistent information to promote patients’ awareness. The patient-physician therapeutic alliance demands transparency in the delivery of information (31) on the diagnostic-therapeutic performance of healthcare institutions.

### Surgical and/or other treatments

Four of the indicators concerned surgical treatments (Nos. 5, 6, 7, and 8), three concerned medical treatments (Nos. 12, 13, and 14), and three (Nos. 9, 10, and 11) addressed multimodal (surgical and medical) therapies.

Of the first four indicators (Nos. 5, 6, 7, and 8), two were entirely consistent with the thresholds. In other words: superficial, small-sized or low-grade STS received appropriate surgical treatment (indicators Nos. 5, 6); surgery for medium- or high-grade STS of the head–neck, trunk or limbs was radical only in 80% of cases (indicator No.7). The fourth, concerning surgery for retroperitoneal STS, was the indicator with the most significant gap between the threshold (>80%). The proportion of patients who actually had multivisceral surgery is only 37%. This result may be (at least partially) explained by three main considerations. One concerns the fact that multivisceral surgery (32–34) has been adopted only recently for the treatment of retroperitoneal STS. Another relates to the fact that retroperitoneal STS accounted for only 50 (23%) of the 214 STS cases considered here. This raises the question of whether this particular care pathway should be critically readdressed or better monitored (to improve the proportion of appropriate treatments), or whether the threshold value should be revised. A third consideration has to do with the costs of treating STS. In a previous study on the same cohort of patients explicitly addressing the costs of STS care, the mean cost associated with a retroperitoneal primary site was twice as high as for any other site for inpatients, and higher for outpatients (€5,144 vs. €712, respectively). Even after allowing for the complexity of primary retroperitoneal STS, these figures recorded in 2017 strongly suggest parallel trends of care inappropriateness and increasing care-costs (35, 36).

The indicators referring to the treatment of large-size STS (Nos. 9 and 10) showed a suboptimal care management (indicator No. 9: threshold >80%, actual practice 35%; indicator No. 10: threshold >80%, actual practice 70%). These significant gaps between the expectations and the results obtained demands a critical reassessment of either the diagnostic-therapeutic procedures implemented or the consistency of the indicators’ thresholds. This need is reinforced by the unsatisfactory values obtained for indicators Nos. 12 and 13, both of which address the timely treatment of large-sized STS after surgery. These data could also be explained by the still high proportion of patients treated not at a referral center in our Region.

### End of life phase

In the terminal phase of their disease (in the 30 days before they died), 31% of the patients considered were given chemotherapy (threshold <10%). This finding prompts both clinical and ethical considerations. The clinical issue concerns prioritizing corrective action to ensure the cost-effectiveness of anticancer drugs, balancing the therapeutic stress and the effectiveness of the clinical advantages. The ethical issue mainly concerns clinicians’ empathy when dealing with patients’ wishes in the most advanced phase of their illness (37–40).

This study has its strengths and weaknesses. The main weakness lies in the small number of cases considered, resulting in a limited number of STS in each diagnostic category. Given the heterogeneous clinicopathological outcome of STS subtypes, this situation can potentially result in significant biases. More extensive studies should address performance in care provision for early vs. more advanced STS, which could be affected by the biological profile of the malignancies considered, inconsistent clinical management, or (even by) a suboptimal choice of performance indicators. The currently available indicators do not include any quality assessment involving molecular testing, or any reference to personalized therapies, though their impact is already crucial to high-standard patient management (6).

The study’s main strength lies in its population-based (not center-specific) design, so it potentially represents the quality of care offered to a large, epidemiologically stable population. Identifying which indicators did or did not reach the (theoretical) thresholds established by our RWG represents a good starting point for critically addressing what has been achieved so far, and what we need to be done as we move forward.

In conclusion, this study emphasizes that the evaluation of care pathways is critical to ensure quality improvement and excellence of care at all times, especially for rare cancers such as soft tissue sarcomas. In this way, procedures in need of corrective action can be identified and new evidence-based goals can be formulated to improve patient management, outcomes, and optimize resource allocation.

## Data availability statement

The raw data supporting the conclusions of this article will be made available by the authors, without undue reservation.

## Ethics statement

The studies involving human participants were reviewed and approved by Veneto Oncological Institute. Written informed consent for participation was not required for this study in accordance with the national legislation and the institutional requirements.

## Author contributions

MRu and ABu: conceptualization and review and editing. ABru, MS, ST, GG, CC, MZ, and PF: data curation. ABu, CC, and ABra: formal analysis. CR and SM: funding acquisition. ABu: investigation. ABu and VB: methodology and project administration. ABu, ABru, CR, and SM: resources. CC: software. MRu, AD, ABo, PB, LD, MRa, CR, and SM: supervision. ABu, MZ, AV, AS, and ABru: writing original draft. All authors contributed to the article and approved the submitted version.

## Funding

This research has received “Current Research” funds from the Italian Ministry of Health to cover publication costs.

## Conflict of interest

The authors declare that the research was conducted in the absence of any commercial or financial relationships that could be construed as a potential conflict of interest.

## Publisher’s note

All claims expressed in this article are solely those of the authors and do not necessarily represent those of their affiliated organizations, or those of the publisher, the editors and the reviewers. Any product that may be evaluated in this article, or claim that may be made by its manufacturer, is not guaranteed or endorsed by the publisher.
